# Emotion regulation difficulties and differences in autism including demand‐avoidant presentations—A clinical review of research and models, and a proposed conceptual formulation: Neural‐preferencing locus of control (NP‐LOC)

**DOI:** 10.1002/jcv2.12270

**Published:** 2024-08-19

**Authors:** Nicky Greaves

**Affiliations:** ^1^ London UK

**Keywords:** autism, demand avoidance, dysregulation, emotion regulation, formulation, intervention, LOC, preferred interests, sensory, social understanding, theoretical model

## Abstract

Emotion regulation (ER) difficulties and differences in autism are well documented in both research and clinical literature, negatively impacting well‐being for autistic young people. Emotion dysregulation can significantly decrease access to opportunities to learn life skills and increase the risk of mental health problems in adulthood. This situation intensifies with more extreme demand avoidant presentations. Efforts to increase understanding in this area have therefore been the subject of much attention with conceptual models created to explore possible underlying mechanisms and guide interventions. This clinical review explores the ER literature and conceptual models in autism and offers a formulation—Neural Preferencing Locus of Control (NP‐LOC). NP‐LOC aims to build on existing theory, research and conceptual models by offering different perspectives in ER through a cognitive, developmental formulation related to the core features of autism—in particular, the impact of a strong need to follow preferred agendas and routines, differences in social understanding related to daily demands, and difficulties accessing social support systems—and how these factors relate to perceived safety and control needed for daily functioning. The role of social understanding as a mediating factor in ER and the implications for intervention in autism are discussed, especially for demand avoidant presentations.

**Key points**

**What's known?**

- Differences and difficulties in Emotion Regulation (ER) are considered ubiquitous to autistic populations and presents a transdiagnostic challenge in terms of improving understanding and intervention.

**What's new?**

- The clinical review explores theory and research literature on ER in autism from which a formulation—Neural‐Preferencing Locus of Control in autism (NP‐LOC)—describes a biopsychosocial developmental pathway that increases the likelihood for daily life events to trigger ER challenges and dysregulation. It suggests intervention strategies and recommends further research into neural, cognitive and behavioural mechanisms underpinning ER difficulties and differences in autism.

**What's relevant?**

- NP‐LOC hypothesises two core impairments may account for greater ER difficulties and differences in autism and promotes active teaching methods to increase the range of functional ER skills, including improving social understanding around daily demands and accessing social support.

## BACKGROUND

Clinical interventions aim to help autistic individuals learn skills to self‐regulate in response to daily stressors. The author is a clinical psychologist of twenty years’ experience working with autistic young people: five years in screening and diagnosis and fifteen years in providing psychological interventions to a large (currently 300 pupils) autism‐specific setting of specialist schools across the age and ability range. The most significant intervention needs are around emotion regulation differences and difficulties, and existing evidence‐based best practice (NICE, 2013) has been used to reduce barriers to learning and increase access to gaining skills that promote well‐being and independence. Whilst the author has considered outcomes helpful there has remained a wish to explore the experience of daily life from the perspective of the autistic young person further, especially for those showing demand avoidant presentations. In addition, because observed symptoms do not map efficiently onto diagnostic categories effectiveness may be hampered. The author has sought to improve understanding regarding the depth and complexity of ER through reviewing current autism research literature and models, and creating a conceptual formulation aimed to offer additional perspectives regarding autistic experiences and help target supportive interventions.

This clinical review explores current theory, research and explanatory models for ER in autism and incorporates relevant wider theoretical concepts. It then draws on this knowledge base to develop a psychological formulation in autism, integrating a broad range of biopsychosocial causal factors to promote a shared understanding of the presenting difficulties impacting daily life across the life span ‐ in line with good practice guidance in clinical psychology (DCP, 2011; HCPC‐UK, 2023).

## PREVALENCE OF EMOTION DIFFICULTIES AND DIFFERENCES IN AUTISM

Most studies on the experience and expression of emotion in autistic spectrum disorder, (hereafter described as autism) have focussed on anxiety. In a meta‐analysis of 83 studies, van Steensel and Heeman (2017) reported that autistic youth showed significantly higher levels of anxiety when measured dimensionally compared to non‐autistic youth in the general population and clinical settings, with greater difficulties in accessing and differentiating emotional responses, and in expressing emotional insight through adequate facial expression and verbal/non‐verbal means.

Higher rates of psychiatric conditions in autistic youth have been found both when viewed as comorbid or as co‐occurring (Joshi et al., 2010; van Steensel et al., 2013). Simonoff et al. (2008) used an adaptive psychiatric interview to distinguish autism symptoms from other Axis I psychiatric symptoms and similarly estimated that 70% of autistic 12‐ to 16‐year‐olds met the criteria for at least one comorbid condition, with anxiety disorders being the most common set of disorders, at 42%.

Anxiety can be defined as autonomic arousal creating nervous tension often accompanied by general distress (Clark & Watson, 1991). From infancy to adulthood, autistic individuals experience lower levels of positive affect, have more experiences of negative emotions and dysregulation, prolonged periods of resignation and avoidance, and problems soothing once aroused (e.g. Garon et al., 2009; Jahromi et al., 2012; Konstantareas & Stewart, 2006; Losh & Capps, 2006; Mazefsky et al., 2012).

These emotional experiences are understood to be automatically triggered by a central representation whereby the autonomic nervous system generates physiological bodily responses activated in the limbic system (In Lange & James, 1922). These responses urge the body to quickly take specific, rigid, and limited cognitive and behavioural actions to re‐establish safety in response to critical incidents, including fear/avoidance (flight) and anger (fight) triggered by situations perceived as unreasonable, dangerous, or unwanted (Bradford Cannon, 2015). These autonomic networks, innervated by the amygdala, are described as relating to basic early humankind’s warning system to perceived threats, with the necessity to quickly regain immediate safety and perceived control in life‐threatening situations through limited actions of running away from or defending against a marauding animal (the critical incident). In the modern world however, limiting responses to simply acting on the autonomic emotion arousal (e.g. anxiety, anger) through avoidant and aggressive (flight/flight), or passive and overly accommodating (freeze/fawn) behaviours can understandably be unhelpful for coping with the demands of daily life and longer‐term expectations thereof: they are not functional nor flexible enough to cope with the requirements of complex societal needs (attending and engaging in school across childhood, and in adulthood applying for and engaging in work, and managing the social demands to access basic and well‐being needs), and can reduce or create significant barriers to learning and leisure opportunities that promote well‐being. Autistic individuals with depression or anxiety show increased levels of noncompliance, aggressive responses, and irritability (Kim et al., 2000; Matson & Nebel‐Schwalm, 2007; Patel et al., 2017; Tantum, 2000). Correlations with anger, depression, and anxiety have been reported with both parent report data (Hurtig et al., 2009) and self‐report (Quek et al., 2012).

Perceived ‘safety’ is arguably synonymous with perceptions of control. Locus of Control (LOC) is theorised to represent a person’s beliefs about their ability to control outcomes in their life through their own actions in three main ways (Rotter, 1966): an internal LOC perceives outcomes to be contingent on personal actions and an external LOC views outcomes as under the control of either external ‘powerful others’ or ‘chance/fate’. An internal locus of control‐based belief system is associated with highest levels of emotional well‐being. A perceived locus of control related to external ‘powerful others’ can also be helpful to well‐being to an extent as a belief in the powerful other can facilitate seeking social support, advice and training. A locus of control located with external ‘fate’ is associated with increased feelings of helplessness. Increasing externality is associated with poorer functioning and psychopathology, including depression (e.g. Twenge et al., 2004). A study by Hope et al. (2018) explored the relationship of LOC to features of Borderline Personality Disorder (BPD), which is characterised by mood instability, identity disturbance and difficulties in impulse control. After controlling for symptoms of depression, anxiety and demographic covariates, they found support for a unique association between external LOC and BPD features. Thus, having a sense of control over feeling emotionally safe and the ability to regulate emotions effectively appears critical to human daily functioning and emotional well‐being. Feeling safe is hence fundamental to human survival and arguably at the core of the ability to cope with and carry out many daily living tasks, as well as manage social structures.

This is illustrated by Wood and Gadow’s model (2010), which includes a range of experiences that are likely to impact perceptions of safety and control: autism‐related stressors (of social confusion; peer rejections and victimisation related to autism symptoms; prevention or punishment of preferred behaviours or interests; and frequent aversive sensory experiences) leads to increased experiences of overall negative affect with a range of experience‐dependent anxiety disorders (social phobia, obsessive‐compulsive disorder, generalized anxiety, or separation anxiety), or depression. They argue these set up a cycle whereby the negative affectivity contributes to more personal distress which is expressed through autism symptoms, and so increases the likelihood of being presented with the range of stressors, hence completing a negative cycle. This model shows the cycle of a broad range of potential stressors that the autistic population may sadly experience and the understandable resulting emotional problems, and they conclude that broader transdiagnostic mechanisms, not limited to ‘anxiety’, are important to understanding and supporting psychopathology in autistic youth.

In conclusion, observed symptoms in autism do not map efficiently onto diagnostic disorder‐specific categories such as anxiety, and underlying mechanisms may lead to any combination of emotion‐related difficulties (Weiss, 2014). Therefore, this clinical review will instead utilise Emotion Regulation (ER), a construct which keeps distinct the experience of an emotion and the regulation of it. Gross and Thompson (2007) consider ER to be a complex, multifaceted, and interactive process, involving an individual’s neurobiology, cognition, behaviour, affect, and context. It involves intentional and automatic attempts to manage affect, as well as internal (acquired) and external (imposed by others) strategies and is viewed functionally, meaning in relation to the degree that it is effective in facilitating goal attainment. Effective ER therefore involves the ability to monitor and modulate these arousal and emotional responses in the service of engaging in adaptive and socially appropriate behaviour.

Their modal model shows five processes of emotion regulation, with each one leading to the next, and where the response modulation affects one’s situation selection or other ER domains (Level 2). Successful emotion regulation, at any one domain, is considered an individual–relational interaction with parents/caregivers, peers, and other authority figures (e.g., teachers) (Level 1). Each process may be “adaptive” or “maladaptive,” depending on any given strategy’s short‐ and long‐term outcomes.

ER is considered a broad, multilevel process, involving inter‐related systems of attention, physiology and neurological processes (e.g. Calkins, 2010). For example, a teenager might manage his experience of exam anxiety (nervous tension and an urge to avoid) by challenging the automatic negative emotional responses to this ‘perceived threat’ using balanced coping thoughts: he may do so through utilising the social support he has received and internalised throughout his youth to generate self‐talk that it’s ‘normal and OK to feel anxious’, that ‘having a go’ will help him get practice at it and hence become easier over time, and so support his final grade and improve life opportunities. This facilitates him in regulating his anxiety effectively to manage the exam and he finds it wasn’t as bad as he feared. A second teenager may instead avoid school on the morning of the exam. A third may act on the feelings of perceived threat triggered by the exam by hitting out and thus quickly dissipating the associated unpleasant adrenaline sensations. Whilst the second and third teenager may feel better on that day than the first, over time the first teenager may feel less anxious about taking exams, and the second and third may find their responses become increasingly life limiting over time. Successful emotion regulation, through the use of adaptive and the disuse of maladaptive strategies, has been implicated as an instrumental act for positive outcomes in children (Garnefski et al., 2006). As illustrated, ER involves the ability to understand and moderate sometimes intense emotional responses, and strategies can modulate or regulate the emotion responses in ER (adaptive) and emotion dysregulation (maladaptive) ways.

ER also involves an individual‐context transactional process (Gross & Thompson, 2007). That is, the development and continued use of ER requires an alignment among a child’s internal capabilities and the provision of ecological resources found in the family, school, and larger community (Weiss, 2014) and an ongoing process of effective ER requires particular child characteristics and external supports (Geldhof et al., 2014). In the above example, this may be the parent or teacher offering support by facilitating the first teenager to ask for help, share his anxious feelings and be supported to use balanced thinking, or the school noticing and applying for Access Arrangements such as rest breaks to help regulate anxiety. Therefore, key individuals are instrumental in promoting successful ER in young autistic and non‐autistic youth. Parents play a critical role supporting children in selecting appropriate situations, scaffolding skills, developing confidence, and modelling appropriate regulation strategies (Gross & Thompson, 2007). Peers and authority figures (e.g. teachers) also play critical roles in influencing the growth and implementation of adaptive ER (Jahromi et al., 2013).

Equally, such social environments can impact the development of emotion dysregulation (Reaven, 2011); Wood et al. (2009). Reaven and Hepburn (2006) and Reaven (2011) describe how parenting behaviours sometimes facilitate the maintenance of anxiety in autistic youth, whereby greater parenting stress predisposes an increased tolerance for their child’s maladaptive strategies (e.g., avoidance) to everyday situations. Across development this reduces exposure and practice of adaptive ER skills, and this loss in developing a repertoire of adaptive strategies may maintain maladaptive strategies and reduce independence and perceived competence.

The individual‐context transactional process seems to align with attachment theory (Bowlby, 1969/1982) whereby the infant is biologically wired to form attachments that initially serve as a survival strategy (a way to regain feelings of safety during critical incidents of ‘perceived threat’) and which then shape the developing child’s emotional and social development in the ways described above. The author would like to posit that placing both attachment theory and the individual‐context transactional process together maps onto a concept of children having a perceived locus of control located in the ‘Powerful Other’ early in childhood that increasingly becomes cognitively internalised into an internal locus of control as they gradually gain experience and skills.

In line with these theories, studies in typical development demonstrate that effective ER improves dramatically during the first few years of life (Calkins, 2010) and is predictive of positive outcomes and better adaptive behaviour and social relationships (Eisenberg & Fabes, 2006). This self‐regulation (effective ER) is correlated with emotional and behavioural engagement in school (Schlesier et al., 2019; Valiente et al., 2007, 2008) and with prosocial peer engagement and acceptance (Valiente et al., 2012). Effective ER coping strategies therefore involve positive reorientation, communication, and remaining motivated and willing to engage in socially purposeful/cultural activities (using distraction only as a temporary measure when needed to help re‐focus back to task). The author considers that this element of ER necessitates abilities in social understanding including an acceptance of the social concepts and factors needed to function in daily life. These involve understanding that ‘what is functional’ will be based on societal needs (e.g. getting up and going into school) rather than personal preferences (e.g. stay at home to engage in preferred activities), with an acceptance/motivation that daily life involves attempting tasks that are effortful and ‘outside your comfort zone’. Seeking, accepting, and engaging in social support provides protective factors to manage the potential ‘perceived threat’ experiences associated with engaging in new/routine daily life demands and thus over time decreasing anxiety through graded exposure and learning adaptive, functional coping strategies.

## ER DIFFICULTIES AND DIFFERENCES IN AUTISM ACROSS THE LIFESPAN

A recent review by Beck et al. (2020) of research studies measuring ER in autism across the life span showed that greater ER impairment was present. They described difficulties in infants of more frequent and long‐lasting negative emotions (irritability) and aggressive behaviours, and/or nervousness and social withdrawal, and in adolescents, of persistent rumination often days after an incident, intense reactions to social rejection, and continued reliance on parents or caregivers for calming (when no longer normative). By adulthood, studies showed approximately 75% of autistic adults in community samples had co‐occurring diagnoses of either depression or anxiety, with ER impairment considered to underlie these problems.

Mazefsky et al. (2013) concluded that ER impairments appear largely ubiquitous in autism, underlying many of the behaviour problems commonly seen in children and adults (Mazefsky & White, 2014). Autistic youth reported using fewer ER strategies and employing them less flexibly (Cai et al., 2018; Khor et al., 2014) with an increased reliance on potentially maladaptive strategies such as rumination, avoidance, and denial (Khor et al., 2014; Mazefsky et al., 2014; Patel et al., 2017). Available data, focussed mostly on verbal samples, has shown an association between greater anxiety and impaired ER (Cai et al., 2018). Impaired ER in autistic individuals has been found to correlate with more problem behaviours (irritability, tantrums, aggression, self‐harm), co‐occurring psychiatric diagnoses, and negative social outcomes (Mazefsky & White, 2014; Samson et al., 2014; Weiss, 2014). Conner et al.’s (2020) large‐scale parent participation study of 1107 community‐based autistic children aged 6–17 years across the cognitive and verbal ability range, used measures previously validated in autism to evaluate the association between ER and anxiety. They found ER impairment to significantly predict elevated levels of anxiety, adding weight to other research suggesting improving ER leads to decreased anxiety in autism (Conner et al., 2020).

## ER IMPAIRMENT AND CORE AUTISTIC FEATURES

Distinguishing core symptoms of autism from comorbid internalizing and externalizing symptoms is particularly challenging as already described, whereby some symptoms may reflect distinct disorders with unique etiologies, and others are likely “co‐occurring” issues related to the etiology of or associated core features of autism (e.g. Ollendick & White, 2012; Samson et al., 2014; Wood & Gadow, 2010). Although findings on the association between these are mixed (White et al., 2014), there is evidence that children with more significant core features of autism have increased ER difficulties (Samson et al., 2014). A recent multimethod investigation of emotion dysregulation in autistic children that included both parent‐reported and lab‐observed child and parent factors found that both autism features and parenting factors were associated with parent‐reported but not lab‐observed dysregulation (Mills et al., 2022). However, this author considers that because the lab task was a computer game, though intentionally frustrating, it may have represented a popular preferred activity for the autistic children, who were aged 8–13 and of average intelligence.

### Impact of autistic social impairment on ER

Frazier et al. (2021) found that social attention is a single dimension of behaviour that represents a strong preference for social stimuli, and is consistent across cultures, stable across age, and stronger in females, with findings that children with developmental disabilities had lower levels of social attention than neurotypical children, and autistic children had the lowest levels. Whilst social communication difficulties are diagnostic to autism, identifying early points of divergence in infancy has been a challenge. A recent prospective, longitudinal study (Bradshaw et al., 2021) utilising a common clinician‐administered communication assessment for infants aged between 9 and 12 months found at 9 months that autistic infants were already exhibiting significantly fewer social and early speech skills than typical peers, with less robust eye gaze, facial expression and sounds to communicate, and lower rates of communication and gesture use. They concluded that the presence and degree of changes in eye gaze, facial expression, gestures and sounds appeared critical for developing social and language abilities. Hadders‐Algra’s review (2022) describes that early atypical and less accurate processing of all sensory modalities may hamper the development of social communication and motor skills and increase repetitive behaviour, and concludes that these early specific signs between 6 and 12 months may relate to the altered dissolution of certain temporary structures in the brain areas already established to be clearly involved in autism.

Beck et al. (2020) argue that social impairments in autism may play a pivotal role in the ER‐anxiety association. ER occurs in the prefrontal and ventromedial prefrontal cortex, including the amygdala (Kross et al., 2009), and these same areas have been implicated in the social impairments found in autistic individuals (Gallager & Frith, 2003). Studies show higher social impairment was associated with higher ER impairment (Samson et al., 2014). Swain et al. (2015) examined the interaction between social motivation, impaired ER, and social anxiety in young autistic adults using self‐ and parent‐report measures and found that decreased social motivation was associated with greater social anxiety, suggesting that social anxiety may dampen social motivation. They concluded that low social motivation can contribute to social avoidance, increased social anxiety and greater social impairment and that social impairment, misreading social cues, and uncertainty in social situations may undermine ER skills.

Engaging in characteristically adaptive ER strategies has been associated with less social impairment among autistic youth (Goldsmith & Kelley, 2018), and ER skills have been correlated with prosocial peer engagement in preschool autistic children (Jahromi et al., 2013) suggesting that early intervention in improving ER skills is important. An early pre‐school intervention—where parents were trained during play sessions to actively provide social responses to their child’s attempts at communication in ways that were on the child’s terms—showed significant improvements in the child’s behaviour and social communication/interaction over time (Green et al., 2010; Pickles et al., 2016).

### Impact of autistic repetitive behaviours on ER

Autism core symptoms such as restricted interests, repetitive behaviours and sensory sensitivities, and insistence on sameness are associated with anxiety in autistic youth (Rodgers et al., 2012; Uljarević & Evans, 2017). In a sample of 56 autistic youth, whilst all core symptom domains were associated with increased ER impairment, restricted and repetitive behaviours were the strongest predictor of ER impairment (Samson et al., 2014). Jasim and Perry (2023) explored how specific repetitive and restricted behaviours and interests may interfere with well‐being and functioning in autistic individuals. They found older children showed higher rates of Ritualistic/Sameness behaviours than younger children and adolescents, and younger and older children showed more Stereotypy than adolescents. Ritualistic/Sameness and Self‐Injurious Behaviour both predicted internalising and externalising behaviours, and Stereotypy predicted internalising behaviour.

### Co‐occurring autistic core features and ER impairment—Extreme demand avoidance

Demand avoidant features appear to be relatively common in autistic children, involving a strong need to follow a preferred agenda and control others, rigid thinking, and resistance to everyday demands (Gillberg et al., 2015). Whilst extreme ‘Pathological’ Demand Avoidance (‘PDA’) first described by Newson in the 1980s (Newson et al., 2003) remains diagnostically debated, O’Nions and Eaton (2020) argue that consideration of an Extreme Demand Avoidance (EDA) profile, or of relevant behaviours, can be helpful as part of an autism clinical formulation and that autism with co‐occurring oppositional defiant disorder (ODD) or disruptive behaviour is often described in international literature.

They furthermore explain the extreme distress, dysregulation, and severe disruption to everyday functioning (aggressive and destructive responses) in response to apparently innocuous requests do not appear logical, hence ‘pathological’. Their research and clinical observations describe these children as experiencing significant sensory processing difficulties, impacting their ability to self‐regulate, alongside fears and phobias, intolerance of uncertainty, and poor understanding and acceptance of social hierarchy making demands seem unfair and thus more aversive. Additionally, parents have described a strong need for control was evident from infancy, and given the extent and long duration of the dysregulated responses they often quickly compromise and adapt around the child. Children with these profiles appear less socially motivated, with a gradual withdrawal from social activities and increasingly socially isolated, with many refusing nursery and school. However, clinically the author has noted that children may choose to attend an occasional social activity when the activity is novel, of interest to them, or perceived to be important for example, Christmas dinner, show & tell, community outing, exam.

### The impact of individual‐context ER factors and autism

Milton (2012) and Davis and Crompton (2021) propose that autistic individuals have problems understanding others, and similarly, others, including parents and caregivers, have difficulties understanding autistic people creating a ‘double empathy problem’. Bolis et al. (2017) argue that this mismatch of interpersonal dynamics—‘dialectical misattunement’—underlies the social and communicative difficulties experienced by autistic people whereby repeated and reciprocal ‘misattunements’ over time lead to ‘increasing divergencies in communication styles and interactions between non‐autistic people and autistic people’ (Davis & Crompton, 2021). The reduced recognition and effective response to an autistic child’s experience of anxiety, discomfort or distress may lead to the child feeling misunderstood and alone, and without the sense of safety that social support can provide.

## CURRENT MODELS FOR ER DIFFERENCES AND DIFFICULTIES IN AUTISM

Mazefsky and colleagues’ model (2013) proposes ER impairments in autistic individuals are related to interactions among many elements: neural circuitry (e.g., abnormal amygdala/prefrontal cortex function and connectivity); psychiatric conditions; greater baseline levels of negative affectivity and hyperarousal; and autism‐related behaviours (e.g., lack of social motivation, awareness of emotion) and cognitive features (e.g., perseveration, rigidity, poor problem solving, and information processing). Due to the many different potential interactions they conclude it is likely that multiple profiles of ER characterise autistic individuals (Mazefsky et al. (2012) and suggest that a framework to organise ER is important for understanding individual differences and determining specific ways to intervene (Mazefsky & White, 2014).

White et al. (2014) agree with this predisposition to ER impairment in autism. Resulting from differences in cognitive functioning, (executive functioning, abstraction, self‐awareness) sensory sensitivities, and biological risks, they propose a developmental framework in which impaired ER can lead to a host of co‐occurring psychopathologies, partially determined by characteristics associated with being autistic. For example, biases in attention, sensory sensitivities, alexithymia, repetitive interests, restricted behaviours and intolerance of uncertainty, and insistence on sameness may promote avoidance of certain situations. These biases may accentuate the relationship between ER impairment and anxiety (Rodgers et al., 2012).

Their model views ER impairment as a transdiagnostic risk factor affected by multiple mechanisms etiologically linked to autism, with anxiety as one possible, likely outcome. The clinical expression of ER impairment in autism is affected by cognitive, social, and behavioural factors.

For demand avoidant presentations, O’Nions and Eaton’s model (2020) provides a framework whereby everyday demands generate anxiety and distress related to fear and dislike of the demand or having to cease a preferred activity. The resulting avoidance cycles then develop to become reinforced and very resistant to change. They recommend that interventions need to be graded exposure‐type approaches: low‐arousal language around demands such as using humour/playfulness, placing less importance on compliance with demands, and a degree of control through offering (limited) choice, whilst also establishing some non‐negotiable boundaries.

Weiss (2014) argues for a developmental transdiagnostic cognitive approach using Gross and Thompson’s model (2007) utilising the five temporally linked domains of a person–situation interaction where emotions can be regulated—situation selection, situation modification, attentional deployment, cognitive change, and response modulation—to present common adaptive and maladaptive aspects of each component. He then provides a detailed case for how it can be examined through the lens of core autistic features. He argues that through employing a transdiagnostic case conceptualization that examines each component, interventions can support an autistic child’s internal skills for ER when confronted with challenges.

Aldao et al., 2016 considers that given both ER and psychopathology are constantly evolving across development the transdiagnostic approach must be linked with a developmental psychopathology framework to understand the role of ER in co‐morbidity and models of ER impairment need to take into account the influence of time and development on the multiple factors (e.g., environmental and biological). According to this model, distal factors (and moderators) influence proximal factors, which subsequently give rise to symptom expression.

## ER FORMULATION‐HYPOTHESIS IN AUTISM

A formulation utilises such research, theories and models outlined above to hypothesise how underlying factors across development may cause and maintain difficulties using a dynamic narrative‐style framework through which the connection between individual characteristics, experiences and behaviours can be understood. Its purpose is explicitly to provide guidance and target interventions. Whilst a formulation usually applies on an individual basis, the author would tentatively like to suggest the usefulness for this purpose given findings that autism can be viewed as a distinct category. Frazier et al. (2023) investigated the categorical versus dimensional structure of autism spectrum disorder and concluded their findings provided strong support for categorical structure corresponding closely to the autism diagnosis.

### Neural Preferencing Locus of Control (NP‐LOC) in autism—A formulation

The NP‐LOC formulation hypothesises that a neurotypical infant has an innate neural preferencing bias towards utilising social sources (responsive parent, caregiver) when feeling unsafe. Thus, when the sensory system has perceived information to be threatening (infant is experiencing new/overloaded sensations) and neuron pathways have transmitted threat cues to activate the amygdala to initiate fear emotions and distress reactions (crying, looking at parent), the ‘powerful other’ (parent, caregiver) responds to the infant with a range of cognitive and behavioural techniques. These include balanced, solution‐focused, self‐affirming words, and socially adaptive and safe actions. The infant perceives these sensory experiences as helpful in challenging the threat cues, mediating and deactivating ‘perceived threat responses’ and activating feelings of safety and well‐being. In this way the infant can be seen as having a predominantly external ‘powerful other’ LOC for managing ER when ‘perceived threat’ is activated. The success of this neural preferencing bias—the attention to and processing of these social sensory experiences for effective recovery from autonomic nervous system reactivity—increases motivation over time both towards engagement with the ‘powerful other’ and in cognitively processing the sensory information provided (words, actions) as helpful to ER. This enables the developing child to internalise these coping skills to self‐soothe, as well as develop the social understanding that ‘powerful others’—seeking advice from those in authority, asking others for help—are also helpful in effective ER. Across development this process continues, and skills are internalised to form a perceived ‘internal’ LOC with effective ER strategies that include internalised social understanding cognitions to cope with the challenges of daily life. The belief that ‘social interactions can be associated with feelings of safety’ is fundamental to feeling in control, and therefore at the heart of the developing child’s motivation to actively seek and learn social skills to support functional relationships, self‐control and perseverance in the societal demands of daily life, contributing to school readiness and adult independence in society.

In autism, however, research suggests core difficulties are present from birth. The NP‐LOC model hypothesises that these core difficulties relate to neural preferencing processes whereby the autistic infant does not have such a strong neurological preferencing bias towards utilising the sensory information provided by the ‘powerful other’ to deactivate ‘perceived threat’ fear responses. Hearing the soothing voice, touch, the vestibular experience of being lifted up and held close, does not set off neural activity to mediate distress in autism as successfully as in typically developing infants.

Whilst autistic infants can seek parent proximity when distressed, the sensory input provided by the ‘powerful other’ is not attended to and processed at a neural level effectively and is therefore not recognised to be beneficial to provide effective recovery from autonomic nervous system reactivity. Instead, this social‐sensory information may increase sensory overload and neuron pathways continue to transmit threat cues to activate the amygdala to initiate fear emotions. In ‘perceived threat’ mode, this reduces the brain’s ability to respond flexibly, and the infant seeks a limited, preferred agenda to regain feelings of control to mediate the distress response.

Therefore, when experiencing new and overwhelming sensory information the neural preferencing in autistic infants results in increased seeking and attending to preferred rituals, routines and interactions to regain feelings of control and safety. A strong focus (attention, motivation and cognition) towards preferred ways to self‐soothe, feel safe, calm, happy and occupied are prioritised. The resulting thinking style also appears to have benefits to human functioning as there is a non‐reliance on ‘powerful others’ and instead a developing a personal control over actions and environments, with possible evolutionary significance and value.

Neurological processes and attention biases towards preferred agendas to develop resources to feel safe reduces access to critical developmental socio‐cognitive processes for learning about ‘powerful other’ social sources of emotional support. Whilst a bias towards a preferred agenda could involve some choice to seek social interaction for enjoyment it is not to the level of being the primary focus as it is in neurotypical development. Socially initiated emotional support skills provided by this external ‘powerful other’ who, for example, labels and validates emotions, offers soothing words, explanations, functional solutions, and a hug are not attended to in the same degree. They are therefore less likely to be associated as salient to mediating distress and do not become internalised to the same degree. The resulting decrease in attentional and processing bias is likely to reduce access to developing social understanding as the full intrinsic value and benefits of the ‘social world’ are not being fully understood or experienced. This limits learning help‐seeking behaviours, the ability to recognise emotion responses in others, and socially driven threats.

In autism, across development, preferred agendas and routines therefore increasingly become associated with feeling safe and calm, and so are experienced by the autistic person as being effective ER coping strategies, providing positive re‐orientation, communication, distraction, and socially purposeful/cultural activities. Over time they become the adaptive goal‐directed and value‐based behaviours of feeling occupied, gaining mastery, happiness, and enjoyment for example, gaming with online community. This makes these behaviours more likely to increase whereas social behaviours may be more likely to be perceived as less rewarding, stressful, or become more associated with need. Repetitive, highly focussed passions and intense interests has evolutionary significance and value.

Therefore, when a ‘powerful other’ makes a demand aimed to help with functioning in daily life, this is perceived by the young autistic child as thwarting their engagement in their perceived adaptive well‐being behaviour, and in addition, without having the social understanding of the reason for the demand, leads to emotionally dysregulated behaviour: an anxious or indeed an understandably angry response. Furthermore, the reduced access across development to processing self‐soothing strategies—recognising and labelling emotions, internalising balanced/flexible thinking, and seeking support—means these are not internalised into beliefs as ‘helpful to mediate ER’.

In school settings, the autistic child’s preferred agendas are not flexible nor adaptive for managing the expected goal‐directed and value‐based daily demands. This sets up a maintaining loop where the young autistic person is increasingly at risk of feeling unsafe when faced with the many social (new and overwhelming) demands of school life, leading to critical incidents: high levels of anxiety, emotion dysregulation, and increasingly at risk of gaining a perceived ‘external fate’ locus of control thinking style in adolescence.

This impacts on the beliefs and assumptions the young autistic person then forms about themselves, others, and the world. Thus, beliefs are formed around following preferred routines, interacting with others in preferred ways, and seeking or avoiding sensory stimuli overload as ways to feel in control, functioning, happy and safe.

Following the formation of these beliefs, the formulation then considers the condition assumptions and rules: the guidelines for living and value judgements that are learned from our society, that is, our moral, cultural, religious, and social influences, leading to ‘if…then…should or must statements’. As such, these are based within the framework of social understanding.

Because neurotypical development involves gaining beliefs that understand the critical benefits of social interaction to feel in control, and safe, these condition assumption and rules from ‘powerful others’ are more likely to be sought, focussed on and absorbed. In so doing this enables access to helpful coping strategies such as seeking social support from a parent/friend and asking an authority figure for help, and these social experiences enable effective ER and socio‐cognitive learning of the necessary behaviours needed to function adaptively in daily life. Such ‘neurotypical coping strategies’ are internalised cognitively into an internal LOC that offers a wide range of coping cognitions which in time generates more flexible and balanced thinking. As the requirement/wish to increasingly interact with the world grows, this type of ‘social‐internal LOC’ can generally cope with typical daily demands, manage demands/negotiate with authority figures, and remain feeling safe and happy enough to function effectively.

In autism, however, given the beliefs of perceived LOC relate to following preferred routines to gain feelings of safety and control, these condition assumptions and rules from ‘powerful others’ are not viewed as helpful in effective ER and are therefore understandably less likely to be attended to and processed. Instead, engaging in preferred activities and routines has become meaningful and reinforcing as they are associated with well‐being and effective ER: feeling occupied, gaining mastery, enjoyment and happiness. Over time these therefore become the adaptive goal‐directed and value‐based behaviour for the young person—the condition assumptions and rules. In so doing, understanding the need to tolerate uncomfortable feelings (unsafe, anxious) when learning new things outside of a ‘comfort zone’ preferred interest, and why society has authority figures (‘powerful others’) that we can negotiate with but ultimately whose demands we need to adhere to over and above preferred interests, become challenging cognitive concepts. In addition, many of the ‘neurotypical’ condition assumptions and rules may directly challenge the existing core beliefs and become perceived threats, for example, authority figures creating uncertainty through changes, or making unwanted or confusing demands (See Figure 1). This amplifies the need in adolescence to seek feelings of safety through accessing familiar routines and preferred interests.

FIGURE 1NP‐LOC Formulation for ER differences and difficulties in Autism.

**Figure:**
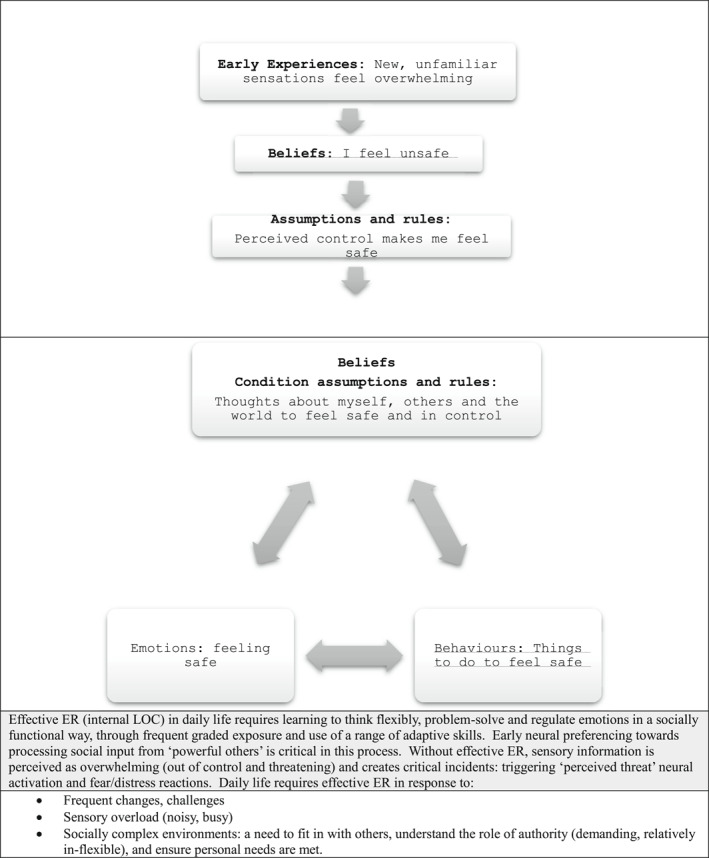

**Figure:**
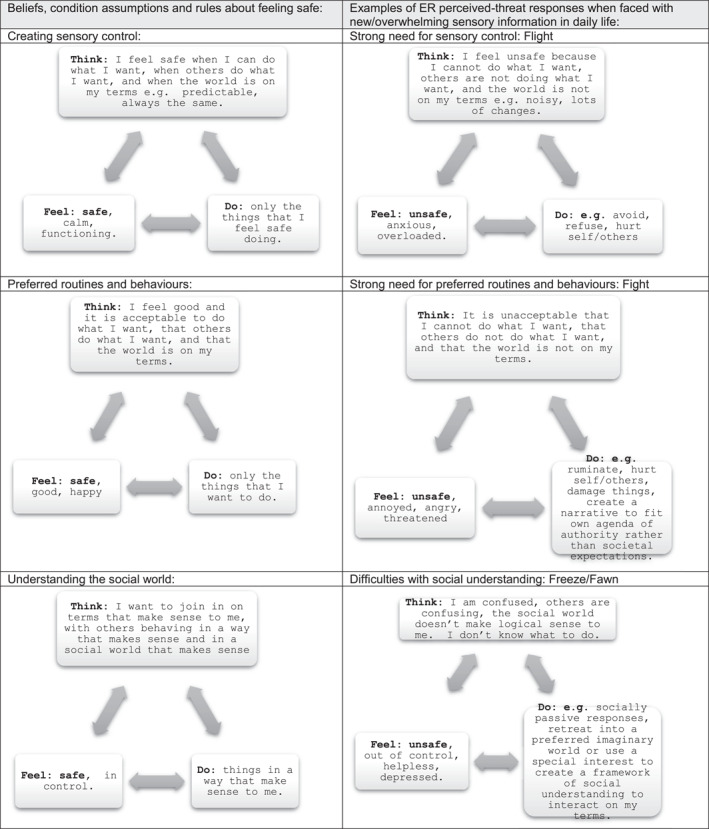

The beliefs and condition assumptions formed through having a neural preferencing and LOC towards a preferred agenda for ER in autism reduces access to socio‐cognitive coping skills. As daily life requires greater flexibility than these condition assumptions and rules allow, daily demands, sensory impact, increasingly complex social interactions can present perceived threats that risk creating ‘critical incidents’. Without having access to the mediating benefits of social support and flexible thinking/action, the critical incidents activate negative, automatic ‘all or nothing’ (extreme) anxious thoughts and the fight/flight/freeze/fawn response. In addition, the core beliefs and condition assumptions for the young autistic person has created the belief that following preferred routines and interests is adaptive, goal‐directive and value‐based, and understandably that engaging in them is therefore acceptable. This is a very different perception of daily functioning than a neurotypical child has gained, leading to the potential for dialectical misattunement.

Therefore, a strong need to control the interaction via fight (challenging behaviour), flight (avoidance) or freeze/fawn (repeated social interactions in preferred or passive ways) may increasingly become coping strategies. These behavioural responses resolve the critical incident in the short term and form ‘coping’ strategies to regain feeling safe, however, they are not functional to many aspects of daily life (Figure 1 illustrates this.).

The high level of social ‘demands’ in daily life therefore increasingly feel unsafe (anxiety‐provoking), challenging, annoying, and confusing for a young autistic person. Without access to a range of socially adaptive coping cognitions, the ‘fight/flight’ response cannot be challenged effectively leading to responses seen in autism such as, avoiding activities, refusing to do activities/becoming angry, using social skills and passive responses with the purpose to avoid social demands, or shifting focus into ‘preferred worlds’ of imagination or preferred activity. However, these strategies do not enable the critical incidents in daily life to be gradually faced in ways that feel safe enough to learn and internalise effective ER and gain internal perceptions of control. As it is very difficult to avoid life’s daily challenges, the continual critical incidents serve to maintain the cycle of ER dysregulation, leading to increased risks of forming a perceived ‘external fate’ LOC for ER and depressive‐type symptoms.

Psychosocial interventions offered from an early age that use adapted sensory low arousal techniques—visual, structured, graded, joined up, and utilise the autistic child’s interests—amplify the experience and psychosocial connection of safety and trust within a social context. In addition, explicit teaching of social understanding skills that include explaining why we have ‘powerful others’ (authority figures) who make demands ultimately to keep us all safe, and why we need to learn to regulate emotions safely for ourselves and others, as well as effective psychosocial help‐seeking strategies, are effective in decreasing anxiety and dysregulated behaviour, increasing flexible responses and improving balanced thinking. They can mediate and protect against critical incidents.

## DISCUSSION

### Clinical review of ER theories and models in autism

The wealth of research on ER and autism has demonstrated that ER impairments in autism present a transdiagnostic challenge. It has enabled a clearer picture of the wide range of presenting emotional and behavioural issues experienced by autistic individuals, and the responses by parents/carers and other authority figures that may impact ER difficulties and differences. Most models have focussed on the significant range of symptoms related to the autism diagnosis that may cause ER impairment. This is effective in bringing cohesion between the different biopsychosocial elements and in showing that interventions need to be flexible enough to account for the full range of responses. However, this situation makes it challenging to establish particular underlying mechanisms that could provide a route to guide targeted interventions.

Many conceptual models have explored the relationship with anxiety only and this has impacted intervention strategies. Beck et al. (2020) argue that despite the varied manifestations of ER impairments in autism and their presence across the life span, most psychosocial treatment research to address emotional problems has focussed on children and the remediation of anxiety and that these protocols generally do not address the other common problems of irritability, anger, and depression. For example, in the demand‐avoidant model O’Nions and Eaton (2020) describe that the EDA child experiences distress (anxiety) at being required to end a preferred activity. However, the author asserts that if one were to experience mastery and well‐being from preferred interests and did not recognise the purpose or benefit to adhere to daily demands, one could arguably expect not just to feel anxious but also to feel justifiably annoyed and irritated should another person ask to stop the preferred task and engage in a seemingly unnecessary and ‘pointless’ demand. This fits with the idea that autistic people may report feeling misunderstood and may understandably hold quite a different view and thinking style to the neurotypical population. An intervention involving a low arousal and graded exposure approach (in line with treating anxiety) may help with distress over loss of control/change generated by the demand. However, if the underlying cognition also relates to difficulties understanding social demands coupled with a strong need to follow a preferred agenda then attempts at graded exposure may equally risk increasing avoidance unless additional support is provided around improving social understanding to ‘see the point’ and benefit of engaging in non‐preferred demands. In fact, a significant proportion of autistic youth receiving intervention for an anxiety disorder continue to exhibit problems with anxiety following the disorder‐specific intervention [Sharma et al. (2021)].

Research and the models described show that ER impairment results from a variety of core processes in autism and may present differently across the life span. Beck et al. (2020) argue that focussing on ER may facilitate effective treatment by addressing multiple behaviours and symptoms simultaneously. They further recommend that interventions that specifically address core ER impairments may have a broader impact over a longer time horizon than treatments focussed on secondary challenges arising from ER impairments, and that clinicians consider treating ER difficulties before engaging in treatment for higher‐level skills, such as social communication or social skills training. This fits well with the models by Weiss (2014), that recommends a structured route to assess specific difficulties in ER, and Aldao et al., 2016 who describes the importance of addressing dynamic change across development. However, these models do not specifically address core impairments and offer strategies, nor recommend which specific areas to target at developmental points across the lifespan.

### Critical review of NP‐LOC

The NP‐LOC formulation hypothesises two core impairments for why young autistic people are at greater risk for experiencing ER difficulties. Firstly, from infancy, the neural mechanisms do not recognise the sensory information (social support from parent/carer) as salient in managing ‘perceived threats’. Instead, beliefs relating to following preferred routines to manage ER are formed, which in turn increases risks to critical incidents being triggered in daily life. Secondly, the resultant lack of skills in seeking social support across development leads to a deficit model, reducing the access to a range of safe and functional ER strategies when responding to a critical incident. As the young person develops, the repetition of this cycle may lead to an external LOC which is associated with increased psychopathology such as depression.

From an ER perspective, NP‐LOC describes why autistic individuals across development may increasingly find preferred routines and rituals important to engage in. Whilst prioritising time in a preferred interest can greatly benefit society, the associated dependence on restricted routines can also lead to greater ER difficulties and behavioural challenges and create a barrier to accessing independence over time. NP‐LOC arguably places ER differences centre stage to provide a formulation for the diagnosis of autism.

NP‐LOC anticipates intervention may initially ‘feel unsafe’ for a young autistic person as their learned reliance for ER is on their preferred agenda rather than socially mediated support and which may feel sensorily overwhelming. Therefore, initial work on building trusting relationships using a focus on preferred interests may be important and require joined up working across school and home settings to ensure unified and consistent messages are being shared. There also needs to be acceptance that, for various reasons, it will take time for this socially adaptive understanding to be processed and learned by the young person. Given childhood provides the developmental window for learning, early intervention appears critical to support this learning and enable greater independence in adulthood. NP‐LOC highlights targeting early psychosocial help through providing safe and enjoyable social interactions, suggesting that amplifying and making explicit consistent, valuing and enjoyable early interactions with the autistic child and their personal interests, and exposure to low arousal (low sensory, visual, structured) shared activities, may improve the development of trust, enjoyment and engagement in healthy social contexts.

NP‐LOC furthermore suggests that given the resultant lack of opportunity to gain effective ER skills, active teaching methods in ER literacy and regulation skills, adapted to age and ability, appear beneficial across the years the young autistic person is in education and into adulthood. One area that the formulation especially highlights is explicit teaching on accessing social support. Practical and logical (i.e. low arousal‐style) approaches involving visual, structured, graded‐exposure techniques that utilise the young autistic person’s preferred interests (NICE, 2013) can be adapted to include offering psychoeducation to understand the brain and bodily responses to ‘perceived threat’, teaching practical skills to support ER literacy and regulation (sensory tolerance, balanced thinking, adaptive self‐soothing, recreational activities), and social skills (sharing worries and asking for help). The aim is to broaden options for adaptive ER to enable resilience.

NP‐LOC suggests that explicit teaching in social understanding appears critical, to include explaining why daily life involves a longer‐term ‘social agenda’ rather than a more immediate individualised ‘preferred agenda’, and why learning to navigate social interaction (including negotiating with authority figures) and engaging in daily demands adaptively and safely is needed for societal functioning. Given the potential for misattunement, providing explanations for ‘how the social world works’—especially the concept of authority figures—may be more successful when made relevant and meaningful to an individual’s interests. Finding ways to share this complex information effectively and support the young person’s social understanding to ‘see the point’ and try tasks they consider ‘beyond their comfort zone’ may be challenging for autistic individuals who experience complex needs and demand avoidance, however, this may be more effective than interventions that only use graded exposure without also supporting social understanding in this way.

There are several limitations for NP‐LOC. A formulation is usually applied uniquely to one individual’s set of circumstances rather than to a diagnostic group. This may reduce the efficacy to apply this hypothesis across the broad breadth of autism presentations, or limit relevance to demand avoidance. Evidence is needed, firstly, to establish this hypothesised neural mechanism in autism and whether ‘sensory‐social’ information is processed in this way; secondly, to verify the experiences of young autistic people to establish beliefs relating to LOC in ER more clearly, as well as any changes across development; and thirdly, to explore social understanding, emotions, and beliefs around daily stressors and social demands, and preferred coping strategies, particularly in more demand avoidant presentations. NP‐LOC formulation does not clearly define which different factors could account for the heightened emotion dysregulation. Heightened sensory sensitivity activating ‘perceived threat’ to reach a critical incident whereby ‘flight‐flight’ activation itself limits flexible processing and responses; or differences with social understanding creating differences in how social support is processed; or a neural preference towards a preferred agenda/difficulties with flexible thinking means social information is not attended to; or indeed aspects of all of these. Each of these would necessitate different clinical interventions making it difficult to target effectively. Finally, interventions need to be trialled that provide additional psychoeducation for social understanding—both around authority‐based demands and in accessing social support—and compared to those with graded‐exposure only.

## CONCLUSIONS

This clinical review explores how ER presents a multi‐faceted, complex challenge in autism, with emotion dysregulation being a transdiagnostic risk factor for psychopathology. These emotional and behavioural problems across the life span can place a huge burden on the autistic individual, family caregivers and service providers, and negatively impact individual and family quality of life. Therefore, gaining an effective biopsychosocial formulation may be helpful to guide targeted interventions to improve well‐being and access independence.

Existing research and models suggest that active teaching of skills to help understand and navigate the psychosocial challenges of daily life as well as creating social (leisure and work) environments that are more accepting, accommodating and valuing of autistic people, appear helpful to improve ER and well‐being. Providing more social support and visual structure, greater flexibility, and access as needed to sensory‐reduced settings can enable autistic people to access and utilise personal ‘functional’ ER to facilitate valuable contributions being made to society.

## AUTHOR CONTRIBUTIONS

**Nicky Greaves**: Conceptualization; formal analysis; investigation; methodology; project administration; validation; visualization; writing — original draft; writing — review & editing.

## CONFLICT OF INTEREST STATEMENT

The author declares no conflict of interest.

## ETHICAL CONSIDERATIONS

Ethical approval was not required as subjects were not involved however ethical considerations were given as part of this clinical review.

## Data Availability

Data sharing is not applicable to this article as no new data were created or analyzed in this study.
